# Over‐Representation of Sperm‐Associated Deleterious Mutations Across Wild and Ex Situ Cheetah (*Acinonyx jubatus*) Populations

**DOI:** 10.1111/mec.70513

**Published:** 2026-08-17

**Authors:** Jessica A. Peers, Heather R. Sibley, Ellie E. Armstrong, Adrienne E. Crosier, Will J. Nash, Klaus‐Peter Koepfli, Wilfried Haerty

**Affiliations:** ^1^ Earlham Institute, Norwich Research Park Norwich UK; ^2^ College of Science George Mason University Fairfax Virginia USA; ^3^ University of California Riverside California USA; ^4^ Center for Animal Care Sciences Smithsonian's National Zoo & Conservation Biology Institute Front Royal Virginia USA; ^5^ School of Biological Sciences University of East Anglia Norwich UK; ^6^ Colossal Biosciences Dallas Texas USA

**Keywords:** cheetah, effective population size, ex situ, inbreeding, male fertility, population genetics

## Abstract

As purifying selection becomes less effective and inbreeding increases, small populations frequently develop an increased load of genome‐wide deleterious mutations. Reflecting this pattern, deleterious mutations in genes associated with fertility and immunity have previously been identified in the cheetah ( *Acinonyx jubatus* ), which has had a low effective population size for at least the last 10,000 years. However, the distribution of deleterious mutations across cheetah populations is currently unknown. Here, we analysed novel whole genome resequencing data from 30 ex situ and 9 wild cheetahs. We investigated variation in genetic diversity, genomic measures of inbreeding, and the distribution of deleterious mutations across cheetah populations. South Sudanese and Tanzanian cheetahs showed higher inbreeding and realised load, while Namibian cheetahs had a higher proportion of population‐specific deleterious mutations. Genes containing high‐ or moderate‐impact deleterious mutations were significantly enriched for sperm‐related functions, highlighting putative causative loci associated with poor sperm quality in cheetahs. Similar levels of genetic diversity and inbreeding were observed in ex situ cheetahs compared to their wild counterparts, providing empirical evidence of the efficacy of captive breeding programmes in maintaining genetic variation in ex situ populations.

## 1 Introduction

Small populations are prone to experiencing increased inbreeding and genetic drift resulting in an accumulation of deleterious mutations (Björnerfeldt et al. 2006; Lande 1994; Masel 2011). In wild populations, this can lead to ‘extinction vortex’ effects, where fixation of deleterious variants and increases in inbreeding continually interact in a feedback loop, resulting in population extinction (Gilpin and Soulé 1986). However, ex situ populations are intensively managed to ensure stable population sizes whilst minimising inbreeding. Despite the potential for such management to mitigate inbreeding effects, ex situ populations often originate from few individuals, resulting in strong founder effects, which decrease genetic diversity and increase inbreeding (Björnerfeldt et al. 2006; Muller 1964). Additionally, in ex situ conditions, natural selection may be relaxed, allowing deleterious alleles to spread through the population (Lynch and O'Hely 2001).

The cheetah ( *Acinonyx jubatus* ) presents an excellent opportunity to study the impacts of ex situ management on a species that has experienced significant inbreeding and prolonged low effective population size (*N*_*e*_). The cheetah is hypothesised to have experienced severe demographic decline beginning 10,000 years ago (Dobrynin et al. 2015; Fabiano et al. 2025; O'Brien and Johnson 2007), resulting in sustained low *N*_*e*_ and phenotypes linked with inbreeding depression, including morphological and skeletal defects, weakened immunity and decreased fertility (Castro‐Prieto et al. 2011; O'Brien et al. 1985; Terio et al. 2018; Wayne et al. 1986; Wildt et al. 1983). Although these phenotypes are typically associated with historical inbreeding, the direct effect of genomic inbreeding on overall fitness in cheetahs remains poorly understood. Currently classified as ‘Vulnerable’ by the IUCN Red List of Threatened Species, approximately 7000 cheetahs remain in the wild (Durant et al. 2017, 2024), with an estimated 2000 cheetahs held in accredited facilities, excluding privately‐owned animals (Marker and Johnston 2022).

The first recorded cheetah in a modern zoological collection appeared in 1829 at the Zoological Society of London, though the first documented ex situ birth occurred in 1956 (Marker‐Kraus 1997). Although ex situ breeding efforts increased substantially starting in the 1970s, including the establishment of a coordinated programme by the Association of Zoos and Aquariums (AZA) in the 1980s, persistent reproductive challenges have limited their success (Marker‐Kraus 1997). Despite an ex situ population approaching 200 individuals, the effective breeding population was estimated at only 28, with infant mortality rates exceeding those observed in other species in ex situ settings (Marker and O'Brien 1989). These reproductive limitations prompted a series of studies revealing low genetic diversity in the cheetah, particularly in MHC genes, as well as poor sperm quality (O'Brien et al. 1983, 1985; Wayne et al. 1986; Wildt et al. 1983).

By the late 1990s, the vast majority of known founders in the global ex situ population originated from the southern African subspecies (*A. j. jubatus*), with minimal representation from eastern Africa (*A. j. jubatus*, previously *A. j. raineyi*) and none from northern African (*A. j. soemmeringii* and *A. j. hecki*) or Asian (*A. j. venaticus*) subspecies (Marker‐Kraus 1997). Over 90% of ex situ cheetahs are believed to descend from wild‐caught Namibian individuals, and although approximately 424 founders were part of the global ex situ population, 80% of those alive in 1994 had not yet bred successfully (Marker‐Kraus 1997).

While founder effects in the ex situ cheetah population may have been mitigated by consistent introduction of wild animals throughout the 20th century, such imports do not guarantee the maintenance of genetic diversity in the ex situ population. Ex situ breeding programmes select breeding pairs based on a minimization of kinship approach, typically informed by a pedigree (Couvet and Ronfort 1994; Fernández and Caballero 2001). However, whilst this practice may reduce breeding between close relatives, it does not necessarily reduce the spread and fixation of deleterious alleles that may already be present in the population due to the generally low *N*_*e*_ of ex situ populations. Low *N*_*e*_ can intensify genetic drift and decrease the efficiency of natural selection, increasing the risk of masked load (heterozygous deleterious mutations) converting to realised load (homozygous deleterious mutations) (Bertorelle et al. 2022; Lewin and Eyre‐Walker 2026; Smeds and Ellegren 2023). Additionally, this method relies on the accuracy of the studbook, which is not well‐maintained in all species (Giontella et al. 2020; Ivy and Lacy 2010). Together, these factors can facilitate the accumulation and spread of deleterious variants within ex situ populations (Woodworth et al. 2002). Furthermore, introducing genetically diverse individuals does not necessarily maintain a population's genetic diversity if those individuals contribute unequally to subsequent generations. For example, if some imported individuals fail to reproduce while others sire many offspring, their genetic contribution to the population becomes skewed, accelerating genetic drift and eroding diversity despite continued introductions.

Cheetahs are known to exhibit signs of poor sperm quality and reproductive defects (O'Brien et al. 1985; Wildt et al. 1983), which may negatively impact reproduction in the wild. However, in ex situ conditions, individuals carrying such mutations may still reproduce, allowing the mutations to persist in the population. For example, in vitro fertilisation (IVF) was carried out on cheetahs in 2020, resulting in two offspring (Crosier et al. 2020). Whilst this use of IVF represents a significant advancement in the application of assisted‐reproductive technologies for cheetah conservation, deleterious mutations which may have otherwise prohibited successful reproduction could be passed to offspring and maintained in the population.

The severe genetic bottleneck and low effective population size of cheetahs predates captivity (Barnett et al. 2005; Kim et al. 2016; O'Brien and Johnson 2007; O'Regan and Steininger 2017), meaning the majority of accumulated deleterious mutations are likely to be shared between wild and ex situ populations. However, these populations have been exposed to contrasting selection pressures and breeding histories. As ex situ breeding programmes often aim to act as insurance or reserve populations for potential rewilding and restoration efforts (Fraser 2008; Witzenberger and Hochkirch 2011), it is important to understand the relationship between the genetic diversity of wild populations and their ex situ equivalents.

Since the first observation of low genetic diversity in ex situ cheetahs (O'Brien et al. 1985), researchers have continued to investigate this phenomenon (Terrell et al. 2016). To date, the majority of studies have relied on microsatellite panels or targeted sequencing approaches such as double‐digest restriction‐site associated DNA sequencing (ddRAD‐seq) (Castro‐Prieto et al. 2011; Driscoll et al. 2002; Meißner et al. 2024; Prost et al. 2022; Terrell et al. 2016). These studies broadly confirm low genetic diversity in cheetahs, although estimated heterozygosity is comparable to or higher than that of other threatened felids such as the Asiatic lion, Eurasian lynx and Iberian lynx (Driscoll et al. 2002; Prost et al. 2022), suggesting the cheetah is not unique among felids in its lack of diversity. While Prost et al. (2022) generated the first genome‐wide insights into genetic diversity in wild cheetah populations using a reduced representation approach, the limited availability of whole‐genome data (*n* = 8) means that genome‐wide patterns of diversity across populations remain largely unresolved (Dobrynin et al. 2015; Winter et al. 2023). In particular, genome‐wide genetic diversity and load have not yet been characterised for any ex situ cheetah population.

Here, we sequenced and analysed whole genomes of 30 cheetahs from the ex situ population in the United States (U.S.) and two wild cheetahs, integrating these with existing genomic data from wild populations to investigate genetic structure, diversity, and the distribution of masked and realised deleterious variants.

## 2 Materials and Methods

### 2.1 Samples

Whole genome re‐sequencing data from 39 cheetahs was used in this study; 32 newly generated and seven from Dobrynin et al. (2015) (Table S1). Of the newly sequenced individuals, 30 were sourced from the U.S. ex situ population across eight institutions (Table S1). Ex situ samples comprised whole blood collected opportunistically in EDTA Vacutainer tubes (Becton Dickinson, U.S.) during routine veterinary exams, except for a single skeletal muscle tissue sample (AJU5640) collected during a post‐mortem necropsy. The International Cheetah Studbook (L. Marker and Johnston 2022) was used to select individuals with minimum mean kinship representing a range of modern lineages: born between 2006 and 2021, with one to nine generations of captive breeding, and a roughly equal split of males and females (16 males, 14 females; Table S1). Several known family groups, selected using the International Cheetah Studbook, were also included as part of a wider study into inheritance of mutation load (Figure S1). The remaining two newly sequenced individuals were collected in South Sudan by Kelsey Greene at African Parks South Sudan (Table S1). The final dataset comprised 30 U.S. ex situ samples, four wild Tanzanian samples, three wild Namibian samples and two wild South Sudanese samples.

### 2.2 DNA Extraction and Sequencing

DNA extraction, library preparation and sequencing for U.S. ex situ samples were conducted at Psomagen Inc. (Maryland, U.S.) resulting in 350‐bp insert libraries (see Supplementary Methods). Libraries were paired‐end sequenced (2 × 150 bp) to 10× coverage on a single 10B flowcell on the Illumina NovaSeq X Plus platform. DNA extraction and library preparation for South Sudan samples were performed by Inqaba Biotec (Pretoria, South Africa) resulting in 150‐bp insert libraries (see Supplementary Methods). Libraries were sequenced on a 25B flowcell on the Illumina NovaSeq X Plus platform at Admera Health (New Jersey, U.S.).

### 2.3 QC, Mapping and Variant Calling

Raw reads (fastq files) were quality checked using *FastQC* v0.11.9 (Andrews 2010). To minimise batch effects, reads from U.S. and South Sudan samples were trimmed to 100 bp to match published samples using *Trimmomatic* v0.39 (Bolger et al. 2014). Adapters were trimmed using *Trim_galore* v0.6.10 with default parameters (Krueger 2015). Paired and unpaired reads were mapped to using *BWA‐MEM* v0.7.17 with default parameters (Li 2013). Read groups were added using *GATK* v4.6.0.0 *AddOrReplaceReadGroups* (McKenna et al. 2010) and BAM files merged and indexed with *SAMtools* v1.18 *merge* and *index* (Danecek et al. 2021).

Variant calling and initial filtering steps were performed using *GATK* v4.6.0.0 (McKenna et al. 2010) (see Supplementary Methods). Variants were further filtered using default *GATK* hard filtering parameters with reduced FS (FisherStrand) requirement (FS < 31.6; 99th percentile) corresponding to our data and minimum and maximum per‐site depth of 119 (1st percentile) and 837 (99th percentile), respectively (GATK Team 2025; Kryvokhyzha 2016). *BCFtools* v1.22 (Danecek et al. 2021) was used to extract biallelic sites, exclude the X chromosome and exclude sites missing in > 15% of samples. Depending on the requirements of each analysis, additional filters were applied using *BCFtools* (Table 1). For some analyses, sites with minor allele frequency (MAF) < 0.025 and related individuals (see below) were excluded. The MAF threshold was chosen to remove rare alleles likely to be sequencing errors. Linkage disequilibrium (LD) pruning was performed with *r* ^2^ of 0.125 and window size of 50,000 kb, again only applied for some analyses (stated below, see Supplementary Methods for full rationale). Finally, invariant sites were removed.

**TABLE 1:** Additional filtering steps applied for each VCF file. All VCFs were filtered using GATK hard filters, biallelic sites extracted and sites missing in > 15% of samples removed. Following this, analysis‐specific filters were applied. Relatives removed: related individuals (AJU10270, AJU10272, AJU9459, AJU9460, AJU9461, AJU9907, AJU9910 and AJU9890) were removed. MAF filter: Sites with minor allele frequency (MAF) < 0.025 were removed. LD prune: linkage disequilibrium (LD) pruning was performed with *r* ^2^ of 0.125 and window size of 50,000 kb. See Supplementary Methods for full rationale for each analysis.

VCF	Relatives removed	MAF filter	LD prune	Number of SNPs	Analyses
1	—	—	—	4,013,408	SnpEff
2	✓	—	—	3,983,129	*F*_IS_, Tajima's *D*, *π*
3	—	✓	—	3,438,984	ROH
4	—	✓	✓	270,848	Kinship, population tree
5	✓	✓	✓	266,132	PCA, ADMIXTURE, *F*_ST_

### 2.4 Kinship

Many population genetics analyses assume unrelated individuals, so kinship among our sample set was assessed to determine if any individuals needed to be removed. *PLINK* v.2.0.0 (Chang et al. 2015; Purcell et al. 2007) was used to calculate kinship using the *KING* algorithm on VCF4 (see Table 1). Results were compared to information from the International Cheetah Studbook (Marker and Johnston 2022) and confirmed expected familial relationships of the known family groups included as part of a wider study. Therefore, for some subsequent analyses (see Table 1), the following related individuals (offspring and full siblings) were removed from the VCF using *BCFtools* v1.22 (Danecek et al. 2021): AJU10270, AJU10272, AJU9459, AJU9460, AJU9461, AJU9907, AJU9910 and AJU9890. Where both parents were included in the analysis, all offspring were excluded. For AJU9889 and AJU9890, the individual with highest genomic coverage (AJU9889) was retained.

### 2.5 Population Structure and Genetic Diversity

To investigate population structure among U.S., Namibian, Tanzanian and South Sudanese cheetahs, a principal component analysis (PCA) was generated on VCF5 (see Table 1) using *PLINK* v2.0.0 (Chang et al. 2015; Purcell et al. 2007) and visualised using the Python package *seaborn* (Waskom 2021). PCAs were also run on all individuals (VCF4), on the U.S. population only, and on the U.S. and Namibian individuals. *ADMIXTURE* v1.3.0 (Alexander et al. 2009) was run on VCF5 to perform ancestry estimation for *K* values 1–5. Pairwise *F*_ST_ values were computed between populations using the Weir and Cockerham estimator (Weir and Cockerham 1984) with *VCFtools* v0.1.16 –*weir‐fst‐pop* (Danecek et al. 2011) on VCF5 in 100 kb sliding windows with a 10‐kb step size. Mean *F*_ST_ values were then calculated across all windows for each pair of populations. To generate a maximum‐likelihood population tree, VCF4 was converted to PHYLIP format using *vcf2phylip* v2.0 (Ortiz 2019) and provided to *RAxML‐NG* v0.8.0 (Kozlov et al. 2019). Model evaluation was performed using *RAxML‐NG* v0.8.0 (Kozlov et al. 2019) to select the model with highest log likelihood, GTR + G, and the tree was generated with 1000 bootstrap replicates. A mitochondrial tree was generated as above (see Supplementary Methods).

To estimate genome‐wide genetic diversity and inbreeding of each population, *VCFtools* v0.1.16 (Danecek et al. 2011) was used to calculate the inbreeding coefficient (*F*_IS_) (Weir and Cockerham 1984; Wright 1965) using –*het*, Tajima's *D* (Tajima 1989) using *–TajimaD* and nucleotide diversity (*π* [Nei and Li 1979]) from variant sites using –*window‐pi* on VCF2. Tajima's *D* and *π* were calculated in 100‐kb windows. Nucleotide diversity (*π*) was also calculated for all callable sites across the genome using *ANGSD* v0.923 (Korneliussen et al. 2014) (see Supplementary Methods). Population‐specific SNPs were identified using *BCFtools* v1.22 (Danecek et al. 2021) and plotted using R package *ggVennDiagram* (Gao et al. 2024). Observed heterozygosity (*H*_*O*_) was estimated for each sample using *ANGSD* v0.923 (Korneliussen et al. 2014) (see Supplementary Methods).

Runs of homozygosity (ROH) were identified from VCF3 using *RZooRoH* (Bertrand et al. 2019) in *R* v4.5.2 (R Core Team 2025). *VCFtools* v0.1.16 (Danecek et al. 2011) was used to remove loci that were significantly out of Hardy–Weinberg Equilibrium (*p* < 0.05), as recommended by *RZooRoH* documentation. A model with multiple homozygous‐by‐descent (HBD) classes was defined using the ‘zoomodel’ function, specifying seven classes with a base rate of 4, and was run on the data. The fraction of the genome in ROH (*F*_ROH_), number of ROH (*N*_ROH_), and total length of ROH (*S*_ROH_) were calculated using ROH > 1 Mb (based on the output of the Viterbi algorithm [Rabiner 1989]) and visualised using *matplotlib* (Hunter 2007). IDrisk (Inbreeding Depression risk), a statistic which multiplies the fraction of the genome in long ROH (here we use *F*_ROH_ > 1 Mb) by the degree of non‐ROH heterozygosity to predict risk of inbreeding depression, was calculated following the method by Kyriazis et al. (2025).

### 2.6 Deleterious Variants

*SnpEff* v5.3 (Cingolani, Platts, et al. 2012) was run with default parameters and a custom database built using the cheetah reference genome (VMU_Ajub_asm_v1.0, GCA_027475565.2 [Winter et al. 2023]) to predict the functional impacts of variants in VCF1. Variants were categorised by predicted impact using *SnpSift* (Cingolani, Patel, et al. 2012). Variants predicted as high‐ or moderate‐impact were extracted and corresponding gene names were identified. From this, *Ensembl BioMart* v115 (Cunningham et al. 2022) was used to extract corresponding human Ensembl gene IDs.

*Orthofinder* v2.5.4 (Emms et al. 2025) was run on the longest protein isoform per gene from the most recent cheetah ( *A. jubatus* ), human ( *Homo sapiens* ), dog ( *Canis lupus familiaris* ) and cat reference genomes (GCA_027475565.2, GCA_000001405.29, GCA_014441545.1, GCA_018350175.1, respectively). One‐to‐one (1:1) orthologues between the human and cheetah were identified and used as the background set for Gene Ontology (GO) enrichment analysis. The foreground set of genes was those with a corresponding 1:1 orthologue and a high or moderate impact SNP. GO enrichment analysis was run for the high and moderate impact SNPs separately, using *ShinyGO* v0.85 (Ge et al. 2020) with an enrichment false discovery rate (FDR) corrected q‐value cutoff of 0.05. Population‐specific SNPs with high and moderate impact were identified using *BCFtools* v1.22 (Danecek et al. 2021) and plotted using R package *ggVennDiagram* (Gao et al. 2024).

Masked and realised load for high‐impact SNPs was characterised per sample by calculating the proportion of all non‐missing high‐impact SNPs per sample that were homozygous alternate (realised) and heterozygous (masked). The same was carried out for moderate‐impact SNPs. Kruskal–Wallis tests were carried out to assess whether each load measure differed significantly across populations, followed by pairwise Wilcoxon rank‐sum tests to identify which populations differed significantly from one another. Both tests were carried out using SciPy Stats (Virtanen et al. 2020) and visualised with *MatPlotLib* (Hunter 2007).

To determine the prevalence of previously identified nonsense mutations across 65 genes (Peers et al. 2025), reads for all samples were mapped to the previous cheetah reference genome, aciJub1 (GCA_001443585.1 [Dobrynin et al. 2015]) using *BWA‐MEM* v0.7.17 (Li 2013) (except Nam4, which is the sample used for this assembly). Read groups were added using *GATK* v4.6.0.0 *AddOrReplaceReadGroups* (McKenna et al. 2010) and BAM files merged and indexed with *SAMtools* v1.18 *merge* and *index* (Danecek et al. 2021). Joint genotyping was completed using *BCFtools* v1.10.2 *mpileup* (Danecek et al. 2021) and the resultant BCF was converted to VCF format using *BCFtools view*. Filters were not applied as depth was calculated separately using *BCFtools query*. The 65 loci previously identified (Peers et al. 2025) were extracted alongside their genotype and per‐sample depth using *BCFtools view* and manually examined to determine prevalence in the population data.

## 3 Results

### 3.1 QC, Mapping and Variant Calling

The total number of paired‐end reads per individual ranged from 103,469,302 to 494,748,773 with a mean depth of 4.2 to 18.5× (Table S2). Mapping efficiency to the reference genome was high, with > 98.9% of reads successfully aligned per individual. 6,896,511 variants were called across 39 individuals. From this, 4,986,770 SNPs were extracted. After *GATK* quality filtering, 4,687,803 SNPs remained, 4,615,749 of which were biallelic. Further filtering of biallelic SNPs to remove the X chromosome and missing genotype data removed 149,510 and 440,852 SNPs, respectively, leaving 4,025,387 SNPs. Depending on the requirements of each analysis, removing relatives, filtering minor alleles and LD pruning steps were carried out (Table 1). When applying all three filters, 266,132 SNPs from 31 individuals remained.

### 3.2 Kinship

**FIGURE 1:**
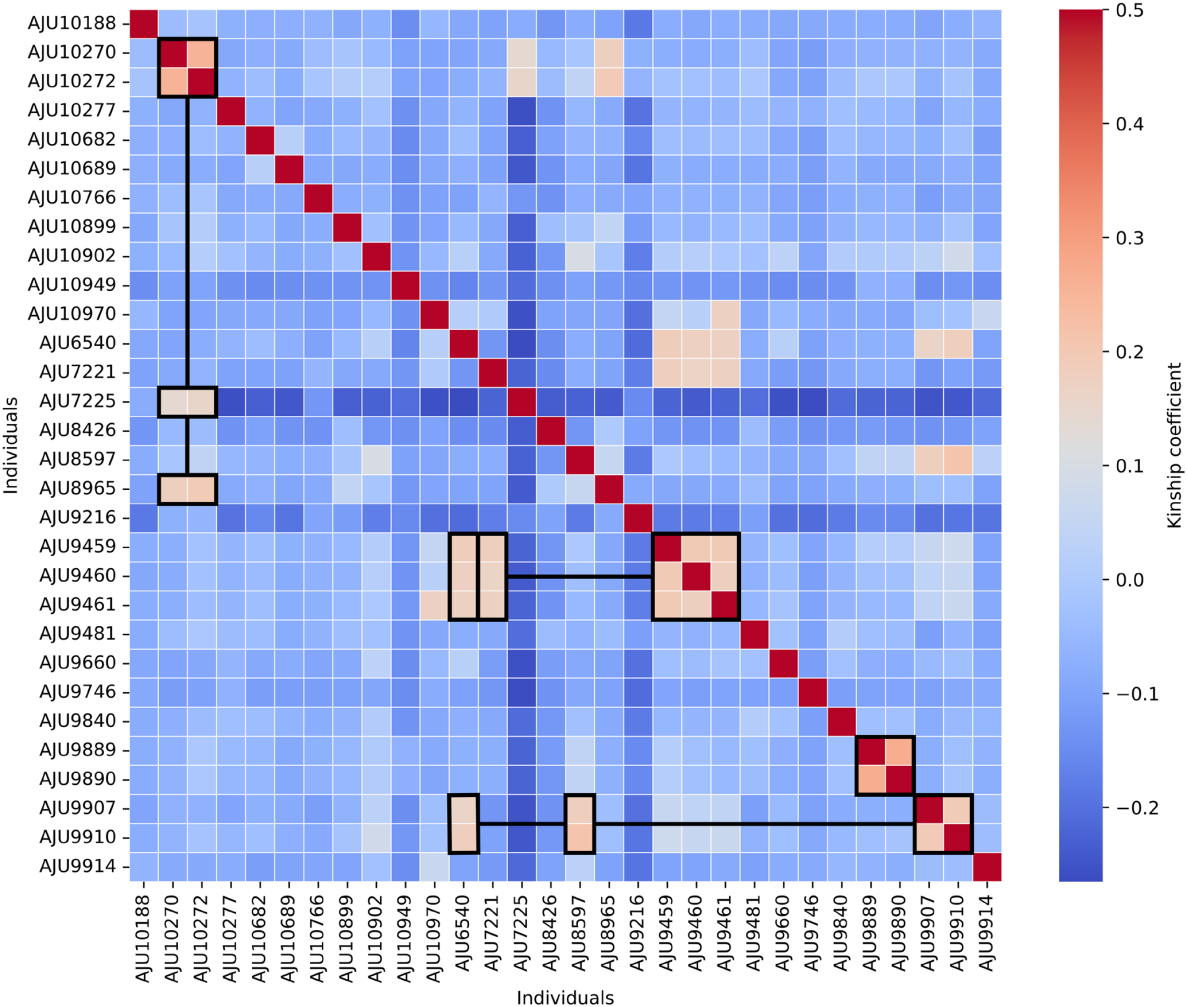
Pairwise kinship values for ex situ U.S. cheetahs. Each cell represents the estimated kinship coefficient between two individuals, with red indicating higher relatedness and blue indicating negative values, suggesting unrelatedness. The diagonal represents self‐relatedness (kinship coefficient = 0.5). Known family groups based on the studbook (see Figure S1) are shown with black boxes and lines connecting offspring and parents. Kinship between most pairs of individuals is low, but first degree family relationships can be observed between known family groups.

Pairwise kinship coefficients were calculated between all individuals and visualised as heatmaps (Figures 1 and S2). Individuals within the U.S. ex situ population had higher kinship compared to the other populations, although Namibian individuals also showed weakly positive kinship to the U.S. population (Figure S2). In comparison, South Sudanese and Tanzanian cheetahs showed lower kinship. Within the ex situ U.S. population (Figure 1), pairwise kinship values were generally low among individuals selected to minimise kinship. Elevated kinship was observed within the known family groups that were intentionally included as part of a wider study, confirming the expected familial relationships recorded in the International Cheetah Studbook (Marker and Johnston 2022). For example, kinship values of 0.25 (suggesting first‐degree relatives) were calculated between AJU10270 and AJU10272, as well as between these individuals and AJU7225 and AJU8965. This is consistent with the studbook record listing AJU10270 and AJU10272 as offspring of AJU7225 and AJU8965.

### 3.3 Population Structure and Genetic Diversity

**FIGURE 2:**
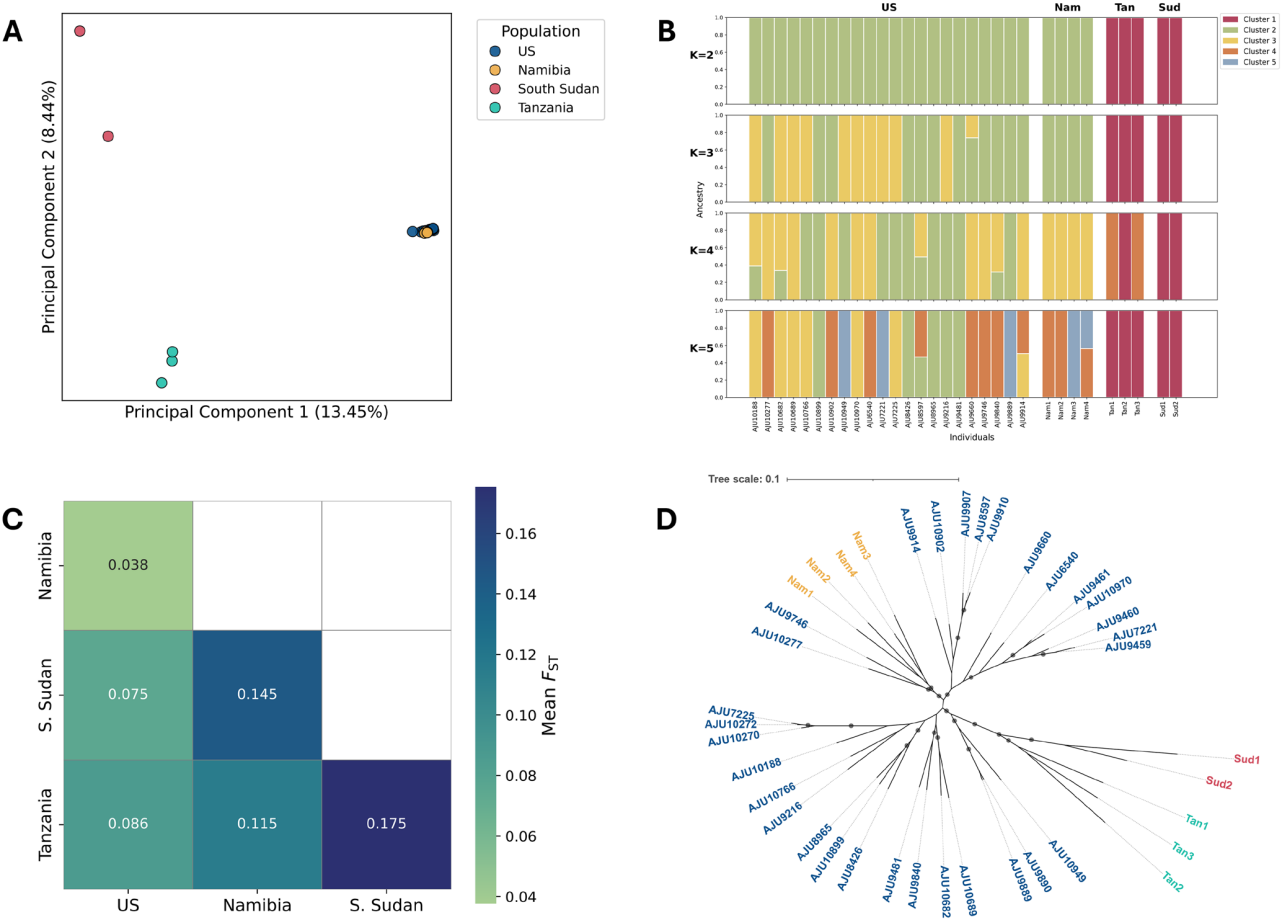
Population structure of ex situ (U.S.) and wild (Namibia, South Sudan, Tanzania) cheetahs. (A) Principal Component Analysis (PCA) of unrelated cheetahs. PCA based on filtered genome‐wide SNPs showing genetic clustering by population: U.S. (blue), Namibia (yellow), South Sudan (red), and Tanzania (green). The percentage of variance explained by each principal component is shown in axes labels. (B) ADMIXTURE analysis of unrelated cheetahs. Model‐based clustering of cheetah populations at *K* = 2–5 using only unrelated individuals. Each vertical bar represents an individual, and colours represent the proportion of ancestry assigned to each genetic cluster. Admixture cross‐validation errors for *K* = 1 to 5 were 0.54, 0.60, 0.69, 0.88 and 0.90, respectively. (C) Pairwise genetic differentiation between cheetah populations. Heatmap showing mean pairwise Weir and Cockerham's *F*_ST_ values among cheetah populations. Darker colours represent greater genetic differentiation, with lighter colours indicating higher genetic similarity. (D) Maximum likelihood phylogenetic tree of cheetah samples based on genome‐wide SNPs. Sample colours match plot A. The scale bar indicates substitutions per site. Branches with bootstrap values > 90% are marked with a grey circle.

Distinct separation was identified between South Sudan, Tanzanian, and Namibian and ex situ U.S. cheetahs (Figure 2A). PC1, separating cheetahs from South Sudan and Tanzania from those in Namibia and the U.S., represented 13.45% of diversity. PC2, which separates the African populations, represented 8.44% of diversity, with remaining PCs representing less than 6% each (Figure S3). A PCA with all individuals, including relatives, showed a consistent pattern (Figure S4). PCAs of the U.S. and Namibian populations (Figures S5 and S6) showed separation of Namibian individuals from the U.S. population, however the variation between U.S. and Namibian individuals was less than that observed within the U.S. population.

Admixture cross validation (CV) errors for *K* = 1 to 5 were 0.54, 0.60, 0.69, 0.88 and 0.90, respectively with the lowest error at *K* = 1, suggesting no population structure (Figures 2B and S7). Genetic differentiation between populations, indicated by pairwise *F*_ST_ values (Figure 2C), mirrored the pattern observed in the PCA (Figure 2A). The U.S. and Namibian populations showed the lowest differentiation (*F*_ST_ = 0.038), suggesting substantial shared ancestry. Greatest differentiation was observed between South Sudan and Tanzania (*F*_ST_ = 0.175), suggesting strong genetic divergence. The population phylogeny based on genome‐wide SNPs mirrored the population structure observed in the PCA and ADMIXTURE analyses, with clear separation of the South Sudan and Tanzanian cheetahs from the U.S. and Namibian individuals (Figure 2D). The separation of South Sudan and Tanzania at *K* = 4, but not at neighbouring *K* values, is likely an artefact of small sample sizes rather than genuine population structure. Bootstrap support was high (> 90) for most branches, particularly those separating South Sudan from Tanzania and the Namibian individuals from those in the U.S. The mitogenome tree showed South Sudanese and Tanzanian cheetahs clustered within the U.S. population, although branch lengths were very short (Figure S8).

**FIGURE 3:**
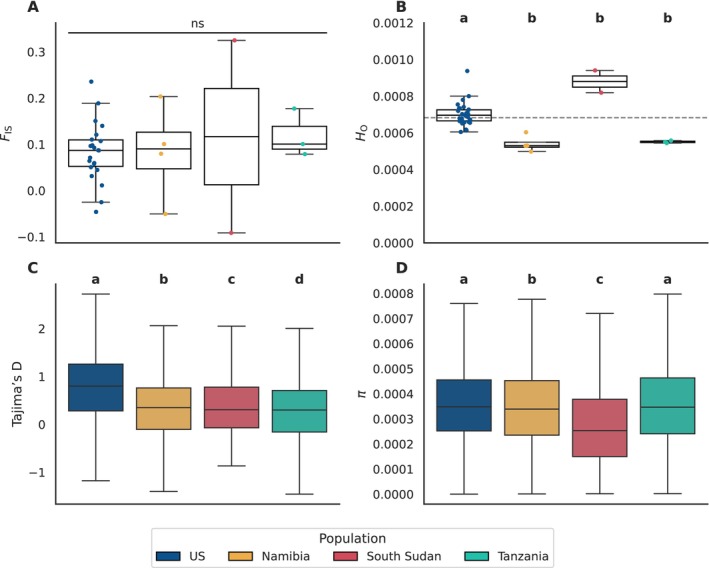
Genetic diversity statistics across cheetah populations. Boxplots showing population‐level estimates of (A) inbreeding coefficient (*F*_IS_), (B) observed heterozygosity (*H*_*O*_), (C) Tajima's *D* (100 kb windows), and (D) nucleotide diversity at variant sites (*π*, 100 kb windows). For all plots, colours indicate populations: U.S. (blue), Namibia (yellow), South Sudan (red), and Tanzania (green), and significant results (Pairwise Wilcoxon rank‐sum tests and Bonferroni correction, *p* < 0.05) are indicated by compact letter display (a, b, c, d) for each plot. See Table S3 for full significance values. In plot B, a dashed line shows the mean *H*_*O*_ across all populations.

Population genetic diversity measures showed higher inbreeding and lower genetic diversity in the South Sudan population, although results should be interpreted with caution given the highly unequal sample sizes across populations, which ranged from 2 to 30 individuals (Figure 3). *F*_IS_ was comparatively lower in the U.S. and Namibian populations (Figure 3A); however, this result was not significant (Kruskal–Wallis: *H* = 0.79, *p* = 0.85). Observed heterozygosity (*H*_*O*_) differed significantly across populations (Kruskal–Wallis: *H* = 20.29, *p* = 1.48 × 10^−4^; Figure 3B), with a global mean of 0.000682 and a range of 0.000498 to 0.000940. *H*_*O*_ was highest in South Sudan and lowest in Namibia and Tanzania, although these pairwise differences were not statistically significant (Bonferroni‐corrected Wilcoxon rank‐sum *p* > 0.05; Table S3), likely due to small sample size (*n* ≤ 4). Tajima's *D* was positive in all populations, consistent with demographic contraction, and was highest in the U.S. population, potentially indicative of a founder effect or population contraction associated with captivity (Figure 3C). A significant overall difference was identified (Kruskal–Wallis: *H* = 6241.81, *p* < 0.001) and pairwise Wilcoxon rank‐sum tests identified a significant difference between all pairs of populations (Table S3). Nucleotide diversity (*π*) at variant sites was significantly different between populations (Kruskal–Wallis: *H* = 4502.52, *p* < 0.001), with significant differences between all pairs of populations except the U.S. and Tanzania (Figure 3D; Table S3). Median *π* ranged from 0.00025 (South Sudan) to 0.00035 (U.S.). Genome‐wide *π*, calculated from all callable sites, was also significantly different between all pairs of populations (Bonferroni‐corrected Wilcoxon rank‐sum *p* < 0.05; Figure S9; Table S3), with median values ranging between 0.00052 (Namibia) and 0.00063 (South Sudan).

**FIGURE 4:**
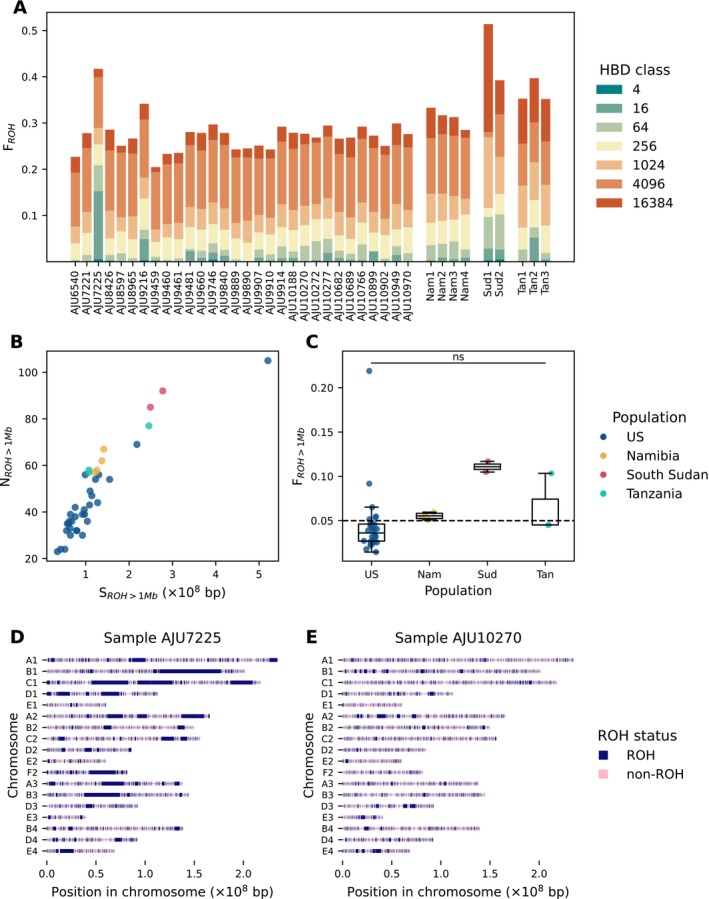
Runs of homozygosity (ROH) across cheetah populations. (A) ROH partitioned into homozygosity‐by‐descent (HBD) classes for each individual. HBD classes reflect approximately twice the number of generations since inbreeding, with more recent ROH (shorter tract lengths) in green and more ancient ROH (longer tract lengths) in red. See Table S4 for mean *F*_ROH > 1Mb_ in long and short HBD classes. (B) The relationship between the number of ROH segments (*N*_ROH > 1Mb_) and the sum of ROH lengths (*S*_ROH > 1Mb_), where colours correspond to populations: U.S. (blue), Namibia (yellow), South Sudan (red), and Tanzania (green). See Table S4 for mean *N*_ROH > 1Mb_ and *S*_ROH > 1Mb_ per population. (C) Fraction of the genome in ROH > 1 Mb (*F*_ROH > 1Mb_) per population, with a data point for each individual overlaid on the boxplot. Colours correspond to populations as described in plot B. Pairwise Wilcoxon rank‐sum tests found no significance (all Bonferroni‐corrected *p* > 0.05), indicated by ‘ns’ label. (D) Distribution of ROH across the genome of AJU7225, where non‐ROH is shown in light pink and ROH is shown in dark blue. (E) Distribution of ROH across the genome of AJU10270, offspring of AJU7225. Colours correspond to those in plot D.

ROH suggested the highest inbreeding in the South Sudan population (Figure 4), with highest mean *F*_ROH > 1Mb_ (0.111, compared with 0.044, 0.055, and 0.065 in the U.S., Namibia, and Tanzania, respectively), and longer ROH compared to the other populations (mean *S*_ROH > 1Mb_ = 263 Mb, compared to 104, 131, and 153 Mb in the U.S., Namibia, and Tanzania, respectively). The South Sudan and Tanzanian populations display a larger proportion of ROH assigned to higher HBD classes (*F*_ROH_ in HBD classes ≥ 1024 was 0.322 in South Sudan and 0.272 in Tanzania, compared with 0.220 in Namibia and 0.201 in U.S.; Table S4), suggesting greater historic inbreeding in these populations (Figure 4A). There were generally fewer and shorter ROH in the U.S. population, with a mean *N*_ROH > 1Mb_ of 41.8 and a mean *S*_ROH > 1Mb_ of 104.1, although several outlier individuals were observed. Individuals AJU9216 and AJU7225 had higher *N*_ROH > 1Mb_ (69 and 105, respectively) and *S*_ROH > 1Mb_ (218 and 520 Mb, respectively) than the rest of the population, and a higher proportion of ROH assigned to lower HBD classes (*F*_ROH_ in HBD classes < 1024 of 0.254 and 0.137, respectively), suggesting more recent inbreeding. Sample AJU7225 showed the highest *F*_ROH > 1Mb_ in the dataset (0.219), with a notably higher proportion of ROH in low HBD classes (*F*_ROH_ in HBD classes ≥ 1024 of 0.254). When visualising the distribution of ROH across the genome (Figure 4D,E), long stretches of ROH can be observed in sample AJU7225 but not in its offspring (AJU10270). Although a Kruskal–Wallis test indicated an overall difference in *F*_ROH > 1Mb_ among populations (*H* = 10.73, *p* = 0.013), no individual pairwise comparison was significant after Bonferroni correction (Wilcoxon rank‐sum tests: all adjusted *p* > 0.05). *F*_ROH > 1Mb_ ranged from 0.014 to 0.211, with a global *F*_ROH > 1Mb_ mean of 0.048. IDrisk scores showed low risk of population decline due to inbreeding depression in all populations (IDrisk < 0.05), based on the score thresholds provided by Kyriazis et al. (2025) (Figure S10; see Box 2 in Kyriazis et al. 2025).

### 3.4 Deleterious Variants

Population‐specific biallelic SNPs were identified in all populations, with most SNPs unique to the U.S. population as expected based on sample size (Figure S11). The Namibian population had the fewest unique SNPs, with over 95% of Namibian SNPs also identified in the U.S. population. Deleterious variants were identified using SnpEff, classifying 598 variants as high impact, 16,952 as moderate impact, 26,525 as low impact and 4,013,328 as modifiers (variant is outside coding regions or its impact is unknown).

**FIGURE 5:**
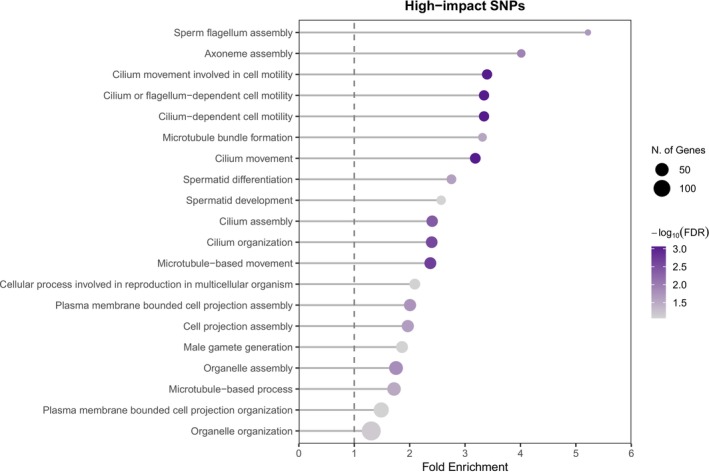
Gene ontology (GO) enrichment of genes with predicted high impact SNPs in cheetahs. GO Biological Process terms significantly enriched (FDR‐corrected *q*‐value < 0.05) among genes containing high‐impact SNPs across the four cheetah populations. Fold enrichment is shown on the *x*‐axis, with the size of the point reflecting the number of genes annotated with each GO term. The colour of each point shows the statistical significance (−log_10_ FDR) of the enrichment of each GO term, where darker points are more significantly enriched. A fold enrichment score of 1, shown by a dotted line, means no enrichment in the gene set.

Six hundred and sixty‐six annotated genes were associated with the 598 high impact SNPs, with some SNPs annotated as affecting multiple genes due to overlapping gene annotations. Of these, 535 had a 1:1 orthologue in the human and so were used as the foreground in GO enrichment analysis. Twenty‐seven Biological Process GO terms were significantly (FDR‐corrected *q* < 0.05) overrepresented in this gene list, mostly related to cilium and sperm flagellum (Figure 5; Table S6).

**FIGURE 6:**
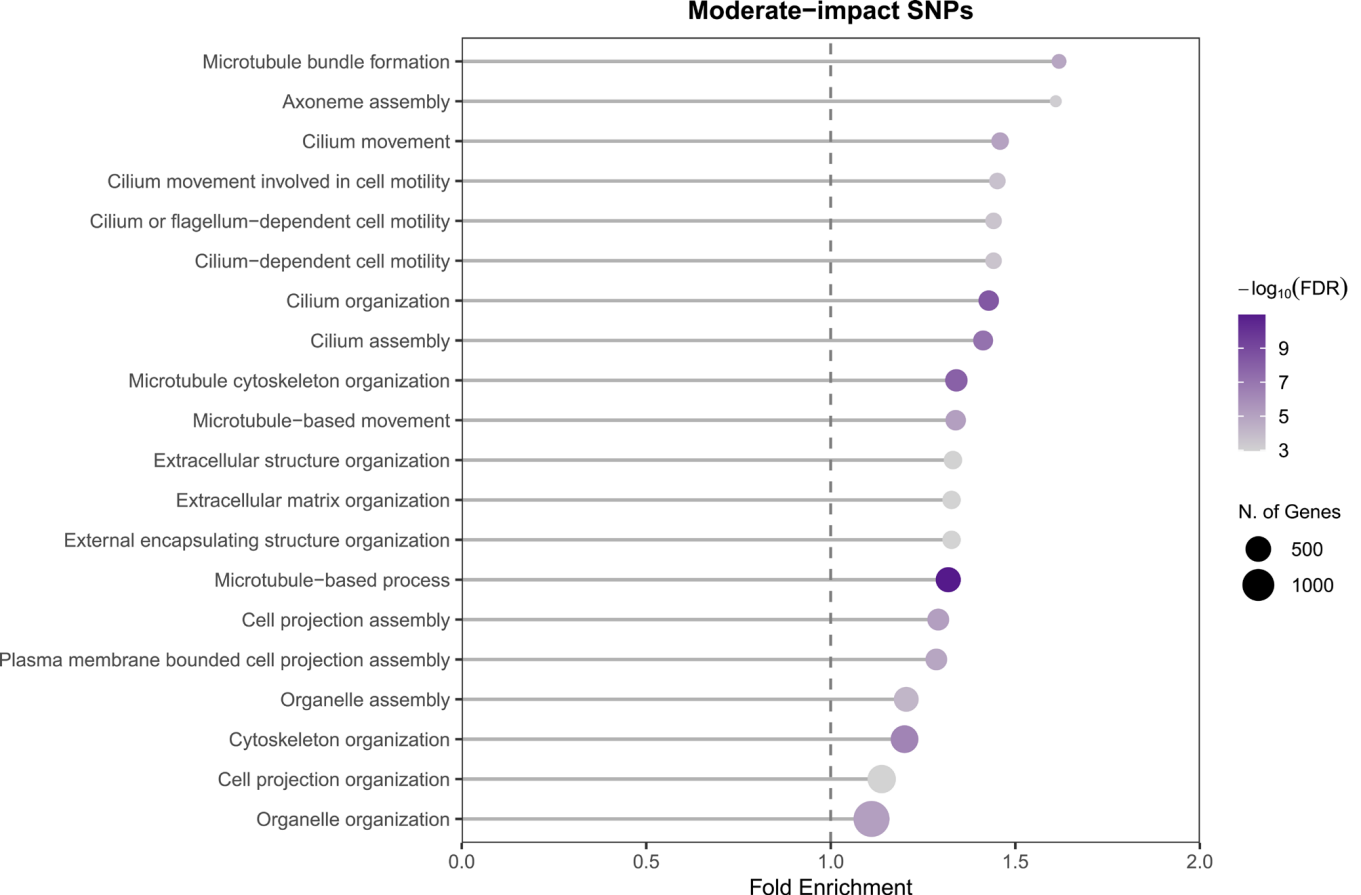
Gene ontology (GO) enrichment of genes with predicted moderate impact SNPs in cheetahs. GO Biological Process terms significantly enriched (FDR‐corrected *q*‐value < 0.05) among genes containing moderate‐impact SNPs across the four cheetah populations. Fold enrichment is shown on the *x*‐axis, point size corresponds to the number of genes annotated with each GO term and the colour of each point shows the statistical significance (−log_10_ FDR) of the enrichment, where darker points are more significantly enriched. A fold enrichment score of 1, shown by a dotted line, means no enrichment in the gene set.

The same analysis was then completed for SNPs with moderate impact. Seven thousand six hundred and ninety‐two genes were associated with the 16,952 moderate impact SNPs. Of these, 6254 had a 1:1 orthologue in the human; this set of genes was used as the foreground for this GO enrichment analysis. One hundred and four Biological Process GO terms were significantly enriched (FDR‐corrected *q* < 0.05) in this gene set. The top 20 most significant (Figure 6; Table S7) were mostly involved in cilium movement and microtubules.

**FIGURE 7:**
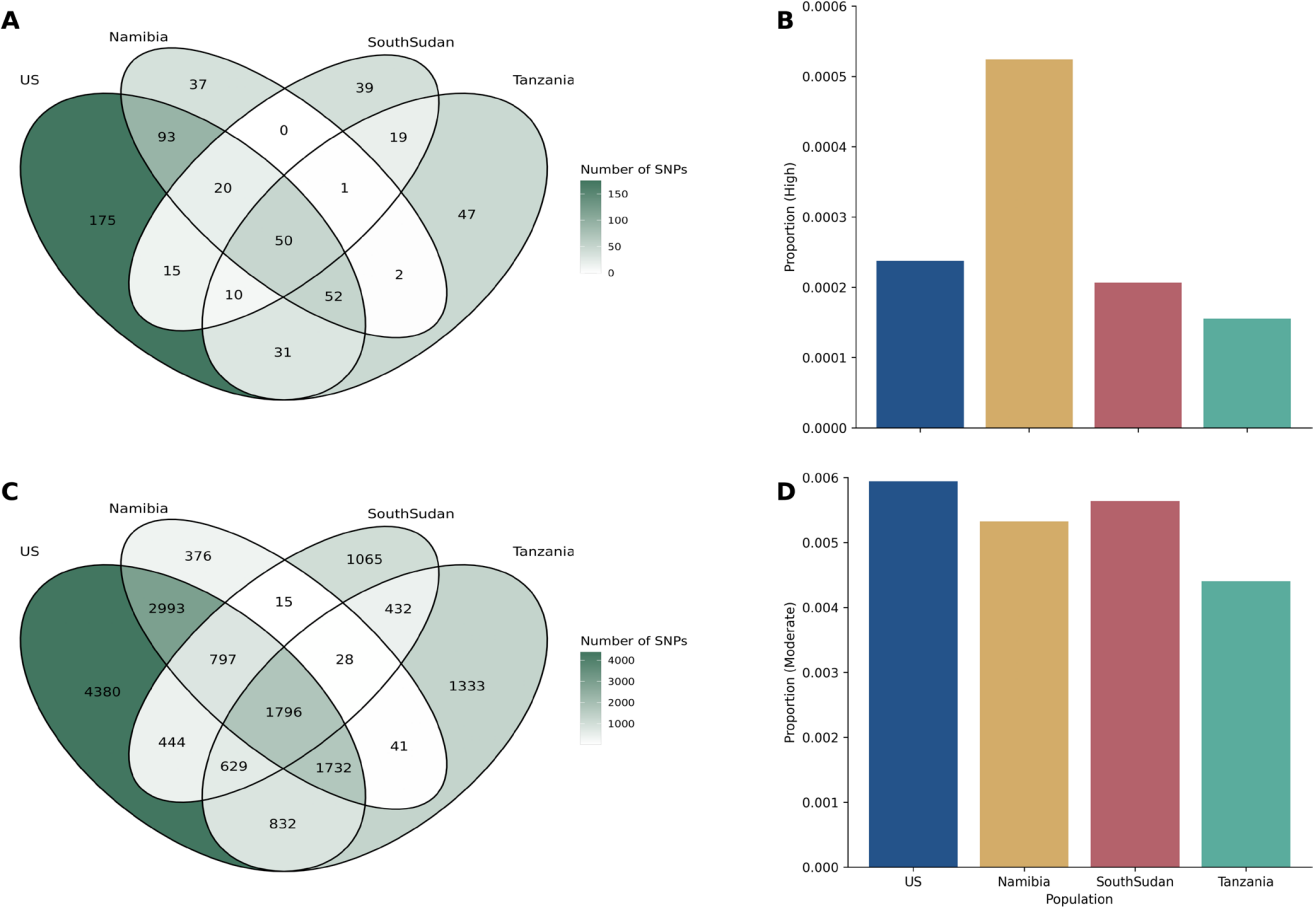
Distribution of predicted high‐ and moderate‐impact SNPs among cheetah populations. (A) Distribution of high‐impact SNPs predicted by SnpEff between the four populations. The number of SNPs specific to each combination of populations is shown in each segment, with a darker colour indicating a higher number of SNPs. (B) Unique high‐impact SNPs as a proportion of the overall number of unique SNPs per population (see Figure S11). Panels (C) and (D) show the same information as panels A and B, respectively, for moderate‐impact SNPs.

High and moderate impact SNPs mirrored that of the total SNP set across the four populations, with each population containing at least 37 unique SNPs with predicted high impact and at least 376 unique SNPs with predicted moderate impact (Figure *7*). When comparing these values to overall numbers of population‐specific SNPs, the Namibian population contained a higher proportion of unique high‐impact SNPs than other populations, whilst Tanzania contained a lower proportion of unique high‐ and medium‐impact SNPs.

The majority of high‐impact SNPs had a low allele frequency (Figure S12), with similar patterns observed in moderate‐ and low‐impact SNPs (Figures S13 and S14). Sixty‐five high‐impact SNPs with a within‐population allele frequency over 0.9 were extracted and their distribution across populations was plotted (Figure S12B). A large proportion of high‐frequency high‐impact SNPs unique to South Sudan was observed, likely due to the small sample size. Additionally, as alleles are not polarised, the high‐frequency high‐impact SNPs may be ancestral. Thirty‐three unique genes were associated with these SNPs. GO enrichment analysis found no significant enrichment (FDR‐corrected *q* > 0.05) of these genes with a high‐frequency, high‐impact SNP.

**FIGURE 8:**
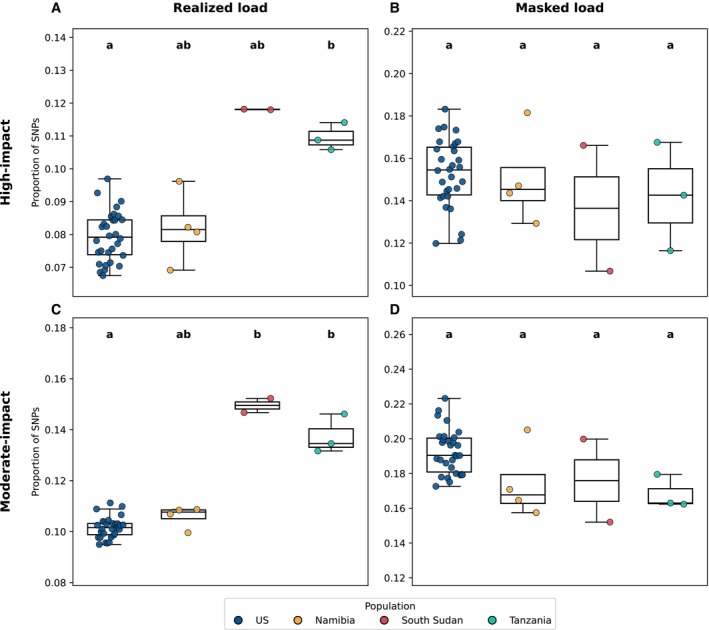
Realised and masked load of high‐ and moderate‐impact SNPs as predicted by SnpEff. For all plots, colours indicate populations: U.S. (blue), Namibia (yellow), South Sudan (red), and Tanzania (green), and significant results (pairwise Wilcoxon rank‐sum tests and Bonferroni correction, *p* < 0.05) are indicated by compact letter display (a, b) for each plot, where populations sharing a letter do not differ significantly. See Table S5 for full significance values. (A) Proportion of homozygous high‐impact SNPs out of the total number of high‐impact SNPs per sample. (B) Proportion of heterozygous high‐impact SNPs out of the total number of high‐impact SNPs per sample. (C) Proportion of homozygous moderate‐impact SNPs out of the total number of moderate‐impact SNPs per sample. (D) Proportion of heterozygous moderate‐impact SNPs out of the total number of moderate‐impact SNPs per sample.

Masked and realised load were assessed per sample as a proportion of total SNPs (Figure 8). A significant overall difference was identified for realised load of both high‐ and moderate‐impact SNPs (Kruskal–Wallis, high: *H* = 12.8635, *p* = 0.00494; moderate: *H* = 14.9022, *p* = 0.00190) and for moderate‐impact masked load (Kruskal–Wallis: *H* = 7.82179, *p* = 0.0498), but not for high‐impact masked load (Kruskal–Wallis: *H* = 1.0992, *p* = 0.777). Bonferroni‐corrected pairwise Wilcoxon rank‐sum tests identified significant differences between both high‐ and moderate‐impact realised load between the U.S. and South Sudan and the U.S. and Tanzania (moderate‐impact only) (Table S5; Bonferroni‐corrected *p* < 0.05). Realised load was highest in South Sudan and Tanzania for both high‐impact SNPs (median proportion of SNPs: South Sudan = 0.118, Tanzania = 0.109, Namibia = 0.081, U.S. = 0.079) and moderate‐impact SNPs (South Sudan = 0.150, Tanzania = 0.135, Namibia = 0.108, U.S. = 0.102), although these differences were not significant (pairwise Wilcoxon rank‐sum tests, Bonferroni‐corrected *p* > 0.05).

Of the 65 previously identified premature termination codons (PTCs) (Peers et al. 2025), 36 were not found in any population data and were therefore considered unique to the reference genome (aciJub1, GCA_001443585.1 [Dobrynin et al. 2015]) as was previously reported (Peers et al. 2025; Table S8). Two loci did not have sufficient coverage to determine population distribution. Twenty‐three loci were homozygous for the reference allele in all individuals, meaning the PTC‐causing mutation is fixed across these samples. The remaining four loci had some variation in the population data. Two were indels, suggesting a false SNP call in the previous analysis. One mutation, in the Four And A Half LIM Domains 5 gene (*FHL5*), was only found in eight ex situ U.S. samples and was otherwise not present in the population data. The final mutation, in the Defensin Beta 116 gene (*DEFB116*), was fixed in all but two ex situ samples, a mother and daughter (AJU8965 and AJU10270).

## 4 Discussion

Understanding the genetic diversity and distribution of masked and realised deleterious mutations across cheetah populations provides insight into the severity of past bottlenecks and their contemporary impact on population health. Here, we present the first whole genome analysis of genetic health of an ex situ cheetah population relative to wild cheetahs. We identify a significant over‐representation of deleterious mutations in sperm‐associated genes, particularly in Namibian cheetahs, whilst Tanzanian cheetahs contained the lowest proportion of deleterious SNPs. We show low genetic differentiation between ex situ and wild Namibian cheetahs, consistent with the proposed origin of the majority of ex situ cheetahs and comparative levels of genetic diversity between ex situ and wild populations.

### 4.1 Population Structure and Relatedness

The genomic evidence presented here corroborates data in the International Cheetah Studbook, as genomic kinship values reflect the familial relationships recorded in the studbook. This is an important result, as ex situ breeding pairs are usually selected based on estimates of minimal kinship from the studbook, which relies on decades of diligent record keeping. Therefore, ensuring that the studbook is accurate is crucial to prevent accidental inbreeding in ex situ conditions (Ballou et al. 2010; Galla et al. 2022).

Population structure analyses consistently showed ex situ U.S. and wild Namibian cheetahs clustering together, reflecting the origin of ex situ cheetahs in the U.S. (Marker‐Kraus 1997). A few individuals from eastern African populations were brought into captivity prior to the 1960s (Marker‐Kraus 1997), which may explain the lower genetic differentiation between South Sudan and Tanzanian populations with the U.S. compared to wild‐wild differentiation. We observed lower *F*_ST_ values across all populations compared to a previous study based on double‐digest restriction site associated DNA (ddRAD) sequencing, despite similar numbers of samples used (Prost et al. 2022). This discrepancy likely stems from the varying resolution of the data types; whole‐genome data can produce more robust estimates of *F*_*ST*_ than reduced‐representation approaches such as ddRAD sequencing (Lou et al. 2021).

Namibian and Tanzanian cheetahs are currently recognised as the same subspecies (*A. j. jubatus*) by the IUCN, although the Tanzanian population was previously classified as *A. j. raineyi* (Krausman and Morales 2005). Based on *F*_*ST*_ estimations, the Namibian and Tanzanian samples used in this study show proportionally high genetic differentiation, supporting Prost et al.'s (2022) call for the IUCN to return to the previous subspecies classification. However, as only four Namibian and three Tanzanian cheetahs were included here, a larger sample set is needed to confirm this observation. Additionally, admixture analysis found no significant separation between samples.

### 4.2 Genetic Diversity and Measures of Inbreeding

Across all sampled cheetah populations, measures of genetic diversity were low, as has previously been observed in the cheetah (Castro‐Prieto et al. 2011; Dobrynin et al. 2015; O'Brien et al. 1983; Terrell et al. 2016). Nucleotide diversity (*π*) values were comparable to previous studies (Castro‐Prieto et al. 2011; Dobrynin et al. 2015); median variant‐based *π* was between 0.00025 and 0.00035, and median whole‐genome *π* was between 0.00052 and 0.00063, supporting the idea first proposed by O'Brien et al. (1983) that cheetahs contain very low genetic diversity. Genome‐wide *π* was significantly lower in Namibia and Tanzania than in the ex situ U.S. population, whereas South Sudan showed significantly higher genome‐wide *π*. In contrast, Namibia and South Sudan had significantly lower variant‐based *π* than both the U.S. and Tanzanian populations. The difference in estimates for the South Sudan population are likely due to the difference in methodologies, as *VCFtools* can underestimate *π* by assuming missing sites are invariant; therefore, the low coverage of Sud2 may have resulted in an underestimate of *π* in this sample (Korunes and Samuk 2021). Regardless, the consistent observation of significantly lower *π* in Namibia supports a previous observation of continued decline in genetic diversity in wild southern African cheetahs (Terrell et al. 2016). Consistent with genome‐wide *π*, observed heterozygosity (*H*_*O*_) was highest in South Sudan, although this result was not significant (Nazareno et al. 2017). All four populations contained higher *H*_*O*_ than previous studies, with a global mean of 0.000682, compared to previous estimations of 0.000400 and 0.000440 (Kim et al. 2016; Prost et al. 2022). Despite this difference, our results support previous observations that cheetahs contain low heterozygosity compared to other mammals (Morin et al. 2021; Pečnerová et al. 2021; Prost et al. 2022). Additionally, positive Tajima's *D* values support the theory that all populations have experienced a genetic bottleneck or population contraction (Dobrynin et al. 2015; Fabiano et al. 2025; Menotti‐Raymond and O'Brien 1993). It is important to note that comparisons between populations with highly unequal sample sizes (ranging from 2 to 30 individuals) should be interpreted with caution, and further sampling of wild populations is necessary to confirm the patterns we observe.

Measures of inbreeding were high across all populations, again supporting previous research (Dobrynin et al. 2015; Menotti‐Raymond and O'Brien 1993; O'Brien and Johnson 2005; Terrell et al. 2016). Median *F*_IS_ was greater than zero in all populations, suggesting all populations have experienced inbreeding. The lack of significant difference in inbreeding between wild and ex situ cheetahs also supports previous observations (Prost et al. 2022). However, as with the previous study, sample sizes for wild cheetah populations remain low, meaning more extensive sampling is required to confirm this finding. Notably, our two South Sudanese individuals showed markedly different *F*_IS_ values, which may reflect distinct individual inbreeding histories or differences in sequencing coverage between the two samples (Sud1: 4.2×, Sud2: 14.2×), and further sampling of this population is required to resolve this. *F*_ROH_ (the fraction of the genome in ROH above a specified length) has often been used to compare inbreeding between populations (McQuillan et al. 2008). Here, we demonstrate that *F*_ROH > 1Mb_ does not significantly differ between cheetah populations. When comparing the distribution of ROH across the genome to previous studies in the cheetah (Dobrynin et al. 2015), we observe far less homozygosity, likely due to differences in the assembly quality of the reference genome (contig N50 = 35.1 Kbp versus 96.8 Mbp for aciJub1 and VMU_Ajub_asm_v1.0 assemblies, respectively) and methodology used to call ROH (Ceballos et al. 2018; Shafer and Kardos 2025; Shi et al. 2026). This is because a more contiguous genome assembly significantly increases accuracy when detecting different size classes of ROH, particularly for longer ROH segments (Shi et al. 2026). Additionally, we observe lower *F*_ROH > 1Mb_ than many other wild and ex situ mammal populations, including those with historic inbreeding and low effective population sizes: Ngorongoro crater lions (Dussex et al. 2025), tigers (Armstrong et al. 2024; Khan et al. 2021), K'gari dingoes (Leon‐Apodaca et al. 2024), Eurasian lynx (Niehaus et al. 2025), Montane red foxes (Quinn et al. 2024), Northern white rhinos (Sánchez‐Barreiro et al. 2021), among others (Brüniche‐Olsen et al. 2018).

Although no significant difference in *F*_ROH > 1Mb_ was observed between populations, South Sudanese cheetahs had higher *N*_ROH > 1Mb_ and *S*_ROH > 1Mb_ than the majority of U.S. and all Namibian and Tanzanian individuals, potentially reflecting more recent inbreeding in this population. However, this observation should be interpreted with caution given the small sample size of these populations, as it may reflect individual‐ or family‐level inbreeding rather than a population‐wide pattern. A greater proportion of ROH in high HBD classes was also observed in the South Sudan population, suggesting this population experienced greater historic inbreeding than the other populations. This population also had a higher proportion of ROH in low HBD classes, suggesting more recent inbreeding, although the highest proportion of ROH in the lowest HBD classes was observed in U.S. sample AJU7225. This individual contains a greater number of ROH and total length of ROH, resulting in the highest *F*_ROH > 1Mb_ in the dataset. This suggests significant recent inbreeding in this individual, also confirmed by its pedigree, which shows several recent cases of inbreeding between half‐siblings (Figure S15). However, as two of AJU7225's offspring were included in this study, it is possible to show that by mating AJU7225 with an individual with lower *F*_ROH > 1Mb_, their offspring can be prevented from inheriting the long ROH observed in their father (e.g., Galla et al. 2022). This finding highlights that recent inbreeding can be reverted provided effective action is taken.

Our data show that the ex situ U.S. cheetah population exhibits comparable measures of inbreeding and genetic diversity to the wild populations, suggesting ex situ breeding programmes are effectively managed to prevent genetic diversity loss, as has been previously suggested (Williams and Hoffman 2009; Witzenberger and Hochkirch 2011). This is strong support for the cheetah ex situ breeding programme, as reduced genetic diversity and increased mutational load (see next section) due to a captivity‐induced founder effect are not observed. With effective management, ex situ populations can act as important reservoirs of genetic diversity and may provide candidates for future reintroduction or genetic reinforcement programmes. However, the success of this approach also depends on factors beyond genetic diversity, including behavioural competence and adaptation to captivity (Crates et al. 2023; Jule et al. 2008).

Future work should apply the whole‐genome resequencing approach used in this study to other major ex situ cheetah populations, such as the European Association of Zoos and Aquaria (EAZA) Ex situ Programme (EEP), to determine whether the genomic patterns observed here are representative of managed populations globally. Such comparisons would provide a comprehensive baseline of genomic diversity across international breeding programmes and could help inform coordinated management and exchange of individuals between ex situ populations.

### 4.3 Deleterious Variants

SNPs with a predicted ‘high’ or ‘moderate’ deleterious impact within protein‐coding genes were significantly enriched for functions associated with male reproductive fitness. Although the relationship between inbreeding and male fertility in cheetahs has been widely debated (Crosier et al. 2018; Fitzpatrick and Evans 2009; Terrell et al. 2016; Wildt et al. 1983), this finding provides strong evidence that cheetahs have experienced an accumulation of deleterious mutations in sperm‐associated genes. This corroborates previous research focusing on premature termination codons and human‐cheetah orthologues of fertility‐related genes, where highly deleterious mutations were identified in several sperm‐associated genes (Dobrynin et al. 2015; Peers et al. 2025). Despite observing high levels of ROH and a relatively high *F*_IS_ in Tanzania compared to the other populations, this population contains the lowest proportion of unique high‐ and moderate‐impact SNPs. Despite this, realised load (homozygous high‐ and moderate‐impact SNPs) is highest in Tanzania and South Sudan, although this result is not significant. This pattern of high realised load is expected in South Sudan, which had higher *F*_ROH_ and therefore higher homozygosity across the genome. This observation may be skewed by low sample size, so future work should aim to sample more individuals from this population. It is also worth noting that SNPs are not polarised, so ancestral and derived alleles are not separated.

When comparing the distribution of these high‐ and moderate‐impact SNPs across populations, we observe a significantly higher proportion of unique high‐impact SNPs related to male fertility in Namibian individuals, although realised load (homozygous deleterious SNPs) is low in Namibia compared to the other wild populations. Comparison of semen samples across wild populations are crucial to assess the impacts of such potentially deleterious SNPs, as previous studies of wild cheetah semen have focused on the southern African subspecies (Crosier et al. 2007; Lindburg et al. 1993; Wildt et al. 1983, 1988). As the majority of ex situ U.S. cheetahs are sourced from the Namibian population, the difference in the proportion of high‐impact, deleterious SNPs between Namibian and ex situ individuals could indicate that these mutations arose more recently in the Namibian population, or that they have been removed from the ex situ population through purging or controlled breeding. As we observed a relatively low proportion of population‐specific high‐and moderate‐impact SNPs in Tanzania, this contrast with the Namibian population may reflect differences in landscape connectivity and land use. Namibian cheetahs predominantly inhabit commercial farmland, fenced game‐farms and protected areas, whereas Tanzanian cheetahs occupy larger, more contiguous ecosystems that may facilitate greater dispersal and increase opportunities for mating between unrelated individuals (Gros 2002; Marker et al. 2003; Marker, Dickman, et al. 2008). These differences may reduce the accumulation and persistence of population‐specific deleterious variants in the Tanzanian population, although further investigation is required to determine the contribution of landscape connectivity to these observed patterns. It is worth noting that population‐specific SNP counts are sensitive to sample size; rare deleterious variants are more likely to be underrepresented in populations with small sample sizes. Despite this potential underrepresentation, the Namibian population still shows the highest proportion of unique high‐impact SNPs, suggesting this observation is robust to sampling bias. Due to the small sample sizes in this study, future work to sequence more genomes from this and other wild populations is necessary to confirm this finding.

It is important to note that the SNP counts of population‐specific mutations are affected by the reference genome used to call SNPs, raising the important issue of reference bias in population genetic studies, which can underestimate genetic diversity and differentiation (Akopyan et al. 2025). As the reference genome used here was generated from an ex situ cheetah housed at Lisbon Zoo, Portugal, we would either expect this bias to falsely reduce the number of unique SNPs in the ex situ U.S. population or in the Namibian population (as the majority of ex situ samples originate from southern Africa), inflating the number of unique SNPs found in South Sudan and Tanzania. Despite this, the U.S. cheetahs still have a higher proportion of unique high and moderate impact SNPs than South Sudan and Tanzania, suggesting that reference bias has not impacted the overall patterns we observe. However, as the origin of the Lisbon Zoo cheetah's wild ancestors was not recorded in the International Cheetah Studbook, it is not possible to confirm the direction of this potential bias.

Our findings have significant implications for cheetah conservation management programmes and integrated conservation strategies (One Plan Approach). Understanding the genomic divergence between wild and ex situ populations is critical for assessing the suitability of captive‐bred individuals for future reintroduction or translocation efforts. Augmentation, a form of translocation whereby individuals are moved between subpopulations to maintain or bolster genetic diversity and reduce inbreeding, has previously been applied to cheetahs in southern Africa (Buk et al. 2018; Marker, Pearks Wilkerson, et al. 2008). Confirming whether the elevated levels of highly deleterious SNPs we observe in Namibia are consistently distributed across the southern African population is necessary to assess any potential threat of facilitating the spread of such mutations. Cheetahs from southern Africa are also being relocated as part of efforts to reintroduce the species to India (Tordiffe et al. 2023; Venugopal 2025). Sourcing individuals from a population with a high mutation load and exposing them to a founder effect could result in these deleterious SNPs becoming exposed through increased homozygosity (Kyriazis et al. 2021; Robinson et al. 2023). Our observation of fewer unique high‐impact mutations in the captive population could support the use of ex situ individuals for reintroduction programmes, although this must be considered cautiously, as previously discussed. Further sampling across the southern African population is necessary to confirm our observation of high mutation load and inform ongoing and future translocation programmes.

Previous research has shown that genomics‐informed captive breeding can reduce inbreeding and mutational load, such as in the pink pigeon ( *Nesoenas mayeri* ) where genetic load was used to identify optimal mate pairs with minimal load in offspring (Speak et al. 2024). In the pink pigeon, this method is more feasible due to the low number of individuals in captivity, making it possible to sample and sequence whole genomes of a larger proportion of the ex situ population. In species with larger ex situ populations, like the cheetah, the time and cost of collecting and sequencing every breeding individual in captivity make this method infeasible. However, identification of deleterious mutations, particularly those impacting key functions like male fertility, segregating at high frequency in the ex situ population could enable the generation of a target‐capture SNP panel that could be used to sequence these loci and prevent fixation of such deleterious mutations through mutational load‐informed breeding recommendations (Bertola et al. 2022; Kleinman‐Ruiz et al. 2017; Wehrenberg et al. 2024).

In conclusion, through resequencing of ex situ and wild cheetah populations, we present the first whole genome comparison of ex situ and wild cheetah populations. We observe low genetic diversity across populations but a more heterozygous genome than other notoriously inbred populations. We confirm the fixation of PTCs in multiple genes associated with male fertility across ex situ and wild cheetahs and identify an over‐representation of deleterious SNPs across sperm‐associated genes, with a high load of these unique to Namibia. Finally, we show that the ex situ cheetah population holds comparable genetic diversity and lower measures of inbreeding compared to wild populations and highlight an empirical example of the use of outbreeding to prevent long ROH being passed to offspring. Our results emphasise the importance of integrating genomic data to inform conservation management of threatened species. Such data are critical for maintaining the adaptive potential of ex situ populations and ensuring that captive‐bred individuals remain genetically compatible with their wild counterparts for future reintroduction.

## Author Contributions

Language used to describe roles below uses the CRediT Taxonomy (credit.niso.org). Conceptualization: W.H., K.‐P.K. Data curation: J.A.P., H.R.S. Formal analysis: J.A.P., H.R.S. Funding acquisition: K.‐P.K., E.E.A., W.H. Investigation: J.A.P., H.R.S. Methodology: J.A.P., H.R.S., W.H., K.‐P.K., W.J.N. Project administration: J.A.P., H.R.S., E.E.A., A.E.C., W.J.N., K.‐P.K., W.H. Resources: E.E.A., A.E.C., W.J.N., K.‐P.K., W.H. Software: J.A.P., H.R.S., E.E.A., W.H. Supervision: W.H., K.‐P.K., W.J.N. Validation: J.A.P., H.R.S. Visualisation: J.A.P., H.R.S. Writing – original draft: J.A.P. Writing – review and editing: J.A.P., H.R.S., E.E.A., A.E.C., W.J.N., K.‐P.K., W.H.

## Funding

J.A.P. was supported by the UK Research and Innovation Biotechnology and Biological Sciences Research Council (BBSRC) Norwich Research Park Biosciences Doctoral Training Partnership [BB/T008717/1]. W.H. was supported by BBSRC grants BBX011070/1 (BBS/E/ER/230001A, BBS/E/ER/230001B, BBS/E/ER/230001C), BBX011089/1 (BBS/E/ER/230002A, BBS/E/ER/230002B), EPSRC EP/X035913/1. The authors also acknowledge support from the BBSRC Core Capability Grant BB/CCG1720/1.

## Ethics Statement

Samples for this study were collected opportunistically (e.g., during routine veterinary practices or necropsies). All ex situ samples were provided with full permission from each zoological facility. This project complied with all Convention on Biological Diversity and the Convention on International Trade in Endangered Species of Wild Fauna and Flora (CBD and CITES) regulations. South Sudan cheetah samples were exported from South Sudan under permit no. 41/2023 and imported into South Africa under CITES permit no. 313932.

## Conflicts of Interest

One author is currently employed by Colossal Biosciences; however, this work was completed prior to that employment. The authors declare no competing interests.

## Supporting information


**Figure S1:** Pedigrees of cheetah family groups included in this study. Females are represented as circles and males as squares. Individuals shaded in grey (AJU8452, AJU7115) were not sampled, but their offspring were included. The dashed line indicates that female AJU6540 contributed offspring to two different family groups.
**Figure S2:** Pairwise kinship values for all cheetahs in this study. Each cell represents the estimated kinship coefficient between two individuals, with red indicating higher relatedness and blue indicating negative values, which suggest unrelatedness. The diagonal represents self‐relatedness (kinship coefficient = 0.5). High kinship is observed within the U.S., Namibia and Tanzania populations and U.S. and Namibian cheetahs share weakly positive kinship. Negative kinship is observed between South Sudan and Tanzanian populations compared to U.S. and Namibian cheetahs.
**Figure S3:** Screen plot showing variance explained by each principal component of a principal component analysis (PCA) run on unrelated cheetahs (Figure 4). Principal components 1 and 2 explain at least 8% of variance each, with subsequent PCs each contributing to < 6% each.
**Figure S4:** Principal Component Analysis (PCA) of all 39 cheetahs used in this study. PCA based on filtered genome‐wide SNPs showing genetic clustering by population: U.S. (blue), Namibia (yellow), South Sudan (red), and Tanzania (green). The percentage of variance explained by each principal component is shown in axes labels.
**Figure S5:** Principal Component Analysis (PCA) of U.S. and Namibian cheetahs. PCA based on filtered genome‐wide SNPs of captive U.S. (blue) and wild Namibian (yellow) cheetahs. The percentage of variance explained by each principal component is shown in axes labels.
**Figure S6:** Principal Component Analysis (PCA) of captive U.S. cheetahs. PCA based on filtered genome‐wide SNPs of US cheetahs. The percentage of variance explained by each principal component is shown in axes labels.
**Figure S7:** ADMIXTURE analysis of all cheetahs in this study. Model‐based clustering of cheetah populations at K = 2–5 using all individuals. Each vertical bar represents an individual, and colours represent the proportion of ancestry assigned to each genetic cluster. Admixture cross‐validation errors for K = 1 to 5 were 0.49, 0.53, 0.55, 0.56 and 0.62, respectively.
**Figure S8:** Maximum likelihood of the mitogenome tree of cheetah samples. Samples are coloured by population of origin: Namibian individuals (yellow), Tanzanian individuals (green), ex situ U.S. individuals (blue), and sequencing replicates (red). The scale bar indicates substitutions per site. Branches with bootstrap values > 90% are marked with a grey circle.
**Figure S9:** Nucleotide diversity (π) per population, calculated using all callable sites. Boxplots show the distribution of π estimated in non‐overlapping 100‐kb genomic windows using ANGSD. Nucleotide diversity was calculated as θπ per callable site (θπ/nSites) from genotype likelihoods. Median values for the U.S., Namibia, South Sudan and Tanzania were: 0.000598, 0.000524, 0.000632 and 0.000528, respectively. An overall significant difference was observed (Kruskal–Wallis: H = 3563.78, *p* = 0), with significant differences between all pairs of populations (Pairwise Wilcoxon rank‐sum tests and Bonferroni correction, *p* < 0.05), as indicated by compact letter display (a, b, c, d). See Table S3 for full significance values.
**Figure S10:** Inbreeding depression risk (IDrisk) for each individual. IDrisk scores combine a measure of the fraction of the genome in ROH (FROH) with the average heterozygosity in non‐ROH regions of the genome. Each individual is coloured based on the severity of the score, with dark red scores showing highest IDrisk. As per the thresholds published by Kyriazis et al. (2025), all individuals show low risk of inbreeding depression. Shapes of plot points correspond to populations: U.S. (circle), Namibia (square), South Sudan (triangle) and Tanzania (diamond).
**Figure S11:** Distribution of biallelic SNPs across the four cheetah populations. The colour of each segment indicates the number of SNPs shared between those populations, with darker green indicating a higher number.
**Figure S12:** Allele frequency of high‐impact SNPs found in protein‐coding genes. A Violin plot showing the allele frequency of all high‐impact SNPs per population. B Distribution of high‐impact SNPs with an allele frequency > 0.9 across the populations. A darker colour indicates a higher number of SNPs.
**Figure S13:** Allele frequencies of moderate‐impact SNPs in protein‐coding genes per population. Allele frequencies were calculated for each SNP classed as ‘moderate’ impact by SnpEff within each population: U.S. (blue), Namibia (yellow), South Sudan (red), and Tanzania (green).
**Figure S14:** Allele frequencies of low‐impact SNPs in protein‐coding genes per population. Allele frequencies were calculated for each SNP classed as ‘low’ impact by SnpEff within each population: U.S. (blue), Namibia (yellow), South Sudan (red), and Tanzania (green).
**Figure S15:** Pedigree of AJU7225 from the ex situ U.S. population. Males are shown with squares and females with circles. Dotted lines indicate individuals that are present in multiple locations across the pedigree. Number IDs for each individual correspond to the International Cheetah Studbook. Individuals with wild origin are denoted with ‘WILD’; all other individuals were born in captivity. Severe inbreeding can be observed between multiple sets of AJU7225's ancestors.

**Table S1:** Sample metadata. Metadata for all cheetah samples used in this study. For all samples, their sex, source population, owner/provider is provided. For relevant samples, their house name, collection date, studbook ID and accession is also provided. Ex situ samples were collected by the NZCBI Veterinary Staff and provided by Dr. Adrienne Crosier and Dr. Klaus‐Peter Koepfli (Smithsonian's National Zoo and Conservation Biology Institute). South Sudan samples were collected by Kelsey Greene (African Parks South Sudan) and sequenced by Dr. Ellie Armstrong at University of California, Riverside. Owner/Providers are as follows: White Oak Conservation, Smithsonian's National Zoo and Conservation Biology Institute (NZCBI), San Diego Zoo, Fossil Rim Wildlife Centre, Wildlife Safari Winston, Toronto Zoo, Toledo Zoo.
**Table S2:** Read quality and mapping. For each sample, the total number of raw sequence reads is provided, followed by the number and percentage of base pairs removed through quality trimming (using Trim_galore) and the remaining read numbers. The number and percentage of reads that mapped successfully (using BWA‐MEM) is shown alongside the average sequencing depth for each individual.
**Table S3:** Significance values for genetic diversity statistics. For each population statistic, a Kruskal–Wallis test was carried out to determine overall significance. H and *p*‐values are provided. For each pair of populations, pairwise Wilcoxon rank‐sum tests were carried out, followed by Bonferroni correction of *p*‐values.
**Table S4:** Mean values for runs of homozygosity (ROH). For each population statistic, the following means were calculated: mean fraction of ROH (FROH) > 1 Mb, mean number of ROH (NROH) > 1 Mb, mean size of ROH (SROH) > 1 Mb, mean FROH > 1 Mb in HBD classes ≥ 1024, and mean FROH > 1 Mb in HBD classes < 1024.
**Table S5:** Significance values for genetic load. For each combination of masked and realised load of high‐ and moderate‐impact SNPs, a Kruskal–Wallis test was carried out to determine overall significance. H and *p*‐values are provided. For each pair of populations, pairwise Wilcoxon rank‐sum tests were carried out, followed by Bonferroni correction of *p*‐values.
**Table S6:** GO enrichment analysis for high‐impact coding SNPs. Gene ontology (GO) enrichment analysis was run using ShinyGO v0.85. The foreground set of genes were those with a predicted high‐impact SNP which also had a corresponding 1:1 orthologue in human. The background set of genes was all 1:1 orthologues between cheetah and human. Results reported are those with an enrichment false discovery rate (FDR) corrected q‐value cutoff of 0.05.
**Table S7:** GO enrichment analysis for moderate‐impact coding SNPs. Gene ontology (GO) enrichment analysis output was run using ShinyGO v0.85. The foreground set of genes were those with a predicted high‐impact SNP which also had a corresponding 1:1 orthologue in human. The background set of genes was all 1:1 orthologues between cheetah and human. Results reported are those with an enrichment false discovery rate (FDR) corrected q‐value cutoff of 0.05.
**Table S8:** Population distribution of previously‐identified PTCs. Sixty‐five previously identified premature termination codon (PTC) loci (Peers et al. 2025) were extracted from the full population VCF using the aciJub1 assembly as the reference genome (Dobrynin et al. 2015). For each locus, the position of the PTC is given, alongside the gene name, its status in the VCF, the reference and alternate alleles, site quality and the number of individuals with each genotype. The final outcome of each locus is given: each mutation is either unique to aciJub1 (the reference), variant in the population or fixed in all individuals sampled. Two sites had insufficient coverage to call.

## Data Availability

Raw sequence reads are available in the European Nucleotide Archive: PRJEB110820. Benefits from this research arise from the sharing of our data on public databases as described above. A collaboration was developed with conservation organisations from South Sudan from which we obtained genetic samples and our research addresses some of their concerns regarding the conservation management of the cheetah.
